# Genome-wide analysis and functional prediction of long non-coding RNAs in mouse uterus during the implantation window

**DOI:** 10.18632/oncotarget.21031

**Published:** 2017-09-16

**Authors:** Qi Wang, Nan Wang, Rui Cai, Fan Zhao, Yongjie Xiong, Xiao Li, Aihua Wang, Pengfei Lin, Yaping Jin

**Affiliations:** ^1^ College of Veterinary Medicine, Northwest A&F University, Yangling, Shaanxi, China; ^2^ Key Laboratory of Animal Biotechnology, Ministry of Agriculture, Northwest A&F University, Yangling, Shaanxi, China

**Keywords:** long non-coding RNAs, RNA-seq, implantation, uterus, mouse

## Abstract

Establishment of the receptive uterus is a crucial step for embryo implantation. In this study, the expression profiles and characterization of long non-coding RNAs (lncRNAs) in pregnant mouse uteri on day 4, day 5 at implantation sites and inter-implantation sites were conducted using RNA-seq. A total of 7,764 putative lncRNA transcripts were identified, including 6,179 known lncRNA transcripts and 1,585 novel lncRNA transcripts. Bioinformatics analysis of the *cis* and *trans* lncRNA targets showed that the differentially expressed lncRNAs were mainly involved in tissue remodelling, immune response and metabolism-related processes, indicating that lncRNAs could be involved in the regulation of embryo implantation. We also discovered that differentially expressed lncRNAs might regulate multiple signalling pathways that play an important role in the regulation of embryo implantation. In addition, nine known lncRNAs and four novel lncRNAs were randomly selected and validated by qRT-PCR. The expression of *Tug1*, *Neat1*, *Gas5*, *Malat1*, *H19* and *Rmst* were significantly regulated in the mouse uterus during the implantation window. Our results are the first to systematically identify lncRNAs in the mouse uterus and provide a catalogue of lncRNAs for further understanding their functions in pregnant mouse uteri during the implantation window.

## INTRODUCTION

In mammalian reproduction, the establishment of a receptive uterus is an essential step for successful embryo implantation, which only occurs in a spatiotemporally restricted time known as the ‘‘implantation window’’. In mice, the endometrium becomes receptive to blastocyst implantation on the morning of day 4 of pregnancy (day of plug = D1). Endometrium receptivity lasts for 18-24 h and usually ends by the afternoon of day 5 of pregnancy [1]. In preparation for implantation, the uterus undergoes a dramatic morphological and functional change, which is regulated by ovarian hormones oestrogen (E_2_) and progesterone (P_4_). In humans, increased implantation failure or early pregnancy loss is observed after implantation occurring beyond the implantation window or at a non-receptive stage [2]. Poor uterine receptivity is considered one of the major causes for the failure of assisted reproductive techniques [3]. In response to the implanting embryo, the receptive uterus generates numerous factors during implantation, but it is revealed that only a few of them are essential to this process. Obtain a global view and identify novel implantation period-specific genes is necessary to further understand molecular mechanisms underlying embryo implantation.

Long non-coding RNAs (lncRNAs) are transcripts longer than 200 nucleotides that lack functional open reading frames and do not encode proteins, usually exhibit lower expression levels, are relatively less conservation across multiple species and exhibit a high degree of specificity among different cell types and tissues [4]. To date, more and more lncRNAs have been identified by microarrays and deep RNA-seq technology. RNA-seq has the potential to capture and identify all of the transcripts expressed in various mouse tissues and cell lines, and could be very helpful in discovering novel lncRNAs unannotated in the current reference genome [5]. Genome-wide studies have shown that similarly to mRNA, lncRNAs are expressed in various tissues and developmental stages [6–9]. Moreover, accumulated evidence has demonstrated that lncRNAs could play critical regulatory roles in many normal physiological processes and various complex diseases, and they have emerged as novel therapeutic targets in tumours [10–12]. The expression profiles of microRNAs in the endometrium of pregnant mice have been well studied as they have major roles in regulating gene expression during embryo implantation [13–15]. Recently, the identification and characterization of lncRNAs in pig endometrial tissue on days 9, 12 and 15 of pregnancy were performed by using RNA-seq [16]. Catalogues of lncRNAs expressed during mouse pre-implantation embryonic development have also been performed using single-cell RNA-seq data [9, 17, 18]. However, to our knowledge, few reports have profiled lncRNAs in pregnant mouse uteri or described how lncRNAs function in embryo implantation.

In this study, RNA sequencing data were integrated from pregnant mice on day 4 (D4U), day 5 at implantation sites (D5IU) and day 5 at inter-implantation sites (D5NU) to obtain a catalogue of lncRNAs in uterus tissues during the implantation window. We further comprehensively identified and characterized the biological functions of differentially expressed lncRNAs through *cis* and *trans* regulated mRNA annotations. Finally, 13 lncRNAs were identified and further validated by qRT-PCR. These results will provide a valuable resource for further understanding the functional roles of lncRNAs during mouse embryo implantation.

## RESULTS

### Genome-wide discovery and identification of lncRNAs in mouse uterus during implantation window

To systematically identify the potential function of lncRNAs in the mouse uterus during the implantation window, uterine samples from pregnant mice on D4U, D5IU and D5NU were collected. Then, transcriptome sequencing was performed using the Illumina HiSeq™ 2500 platform. An overview of the analysis pipeline is shown in Supplementary Figure 1. Approximately 47-51 million raw reads for each sample were produced (Table 1). After removing adaptor reads, sets where poly N comprised more than 10% of reads, and low-quality reads, 97.38-98.22% of clean reads among raw reads in each sample were obtained and used in the following analysis. The GC content of each sample was between 49.86% and 51.14%. Subsequently, approximately 81.39-89.68% of the total clean reads could be mapped to the mouse reference genome sequence using TopHat v2.0.9. The different gene subtypes of the above mapped reads are shown in Supplementary Figure 2 and are based on genomic overlap with existing annotations using the HTseq programme. A total of 274,470 assembled transcripts were produced using both Scripture (beta2) and Cufflinks (v2.1.1). After basic filtering and coding potential filtering, a total of 7,764 putative lncRNA transcripts were identified, including 6,179 transcript isoforms (4,677 known lncRNAs) from GENCODE and 1,585 transcript isoforms (1,298 novel lncRNAs) from the RNA-seq transcript assemblies (Supplementary Table 1).

**Table 1:** Summary of read filter and alignment

Sample	Raw reads	Clean reads	Clean bases	Error rate (%)	GC content (%)	Total mapped
**D4U**	49,778,390	48,892,317 (98.22%)	6.11G	0.04	51.14	43,847,003 (89.68%)
**D5IU**	51,620,615	50,270,636 (97.38%)	6.28G	0.03	50.44	40,912,719 (81.39%)
**D5NU**	47,152,250	46,037,693 (97.64%)	5.75G	0.03	49.86	39,827,875 (86.51%)

### Comparison of features between lncRNAs and protein-coding genes

**Figure 1:**
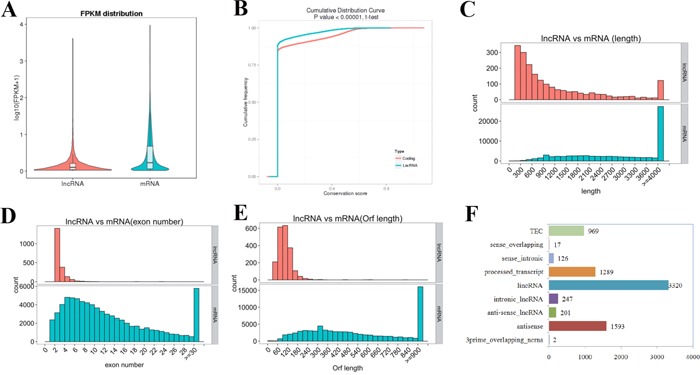
LncRNA characteristics in mouse uterus during the implantation window **(A)** Expression level (FPKM) comparison between lncRNA and protein-coding genes. The FPKM distribution of lncRNAs in mouse uterus is lower than that of protein-coding genes. **(B)** Conservation analysis was evaluated using phyloP (http://compgen.bscb.cornell.edu/phast/). The level of conservation of lncRNAs in mouse uterus is lower than that of protein-coding genes. **(C)** Distribution of transcript lengths in the lncRNAs and protein-coding genes. Transcript size distributions of lncRNAs is generally shorter than that of the protein-coding genes. **(D)** The exon number in lncRNAs and protein-coding genes. 88.41% of the lncRNAs contains two to four exons, while the majority of protein-coding genes consist of more than 10 exons. **(E)** The number of ORFs identified in the lncRNAs and protein-coding genes using Estscan. As expected, the ORFs of lncRNAs is substantially shorter than that of protein coding genes. **(F)** Subtypes of the putative lncRNAs according to the latest gene/transcript biotypes in GENCODE & Ensembl.

Generally, quantification analysis based on the fragments per kilobase of exon per million fragments mapped (FPKM) values demonstrated that lncRNAs in the uterine sample have a lower expression level compared to that observed in protein-coding genes (Figure 1A). As expected, the conservation of lncRNAs in the uterine sample is also substantially lower than what was observed for protein-coding genes (Figure 1B). However, the lncRNA transcript length was mostly within a range of 200 to 800 bp, which was significantly shorter than that of the protein-coding genes (Figure 1C). In addition, significant differences in the distribution of exon number between protein-coding genes and lncRNAs were also observed, and 88.41% of the total lncRNAs only contained two to four exons (Figure 1D). Furthermore, most of the lncRNAs contained relatively shorter open reading frames (ORFs) in comparison to protein-coding genes (Figure 1E). The comparative analysis of the above features between the two transcript species in this study was consistent with that of previous reports.

### Classification of lncRNAs

The 7,764 putative lncRNA transcripts were divided into 3,320 (42.76%) large intergenic non-coding RNAs (lincRNAs), 247 (3.18%) intronic lncRNAs, 201 (2.59%) anti-sense lncRNAs, 1,289 (16.60%) processed transcripts, 1,593 (20.52%) antisense and 969 (12.48%) TEC according to the latest gene/transcript biotypes in GENCODE & Ensembl (Figure 1F). Additionally, a significant difference in gene features was observed among the lncRNA subtypes. The average length of intronic lncRNAs was longer than that of other types of lncRNAs, but the average number of exons and length of ORFs were lower (Supplementary Table 1).

### Differentially expressed lncRNAs in the mouse uterus during the implantation window

**Figure 2:**
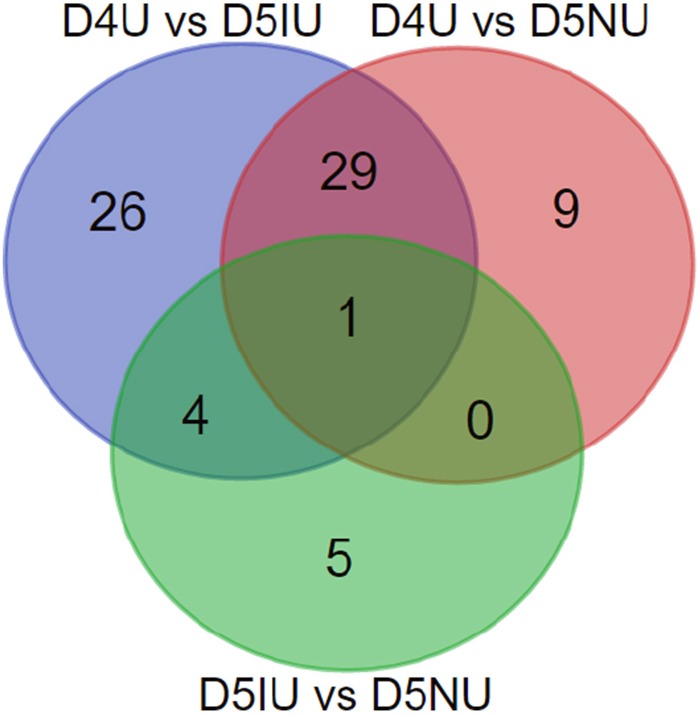
Venn diagrams of the differentially expressed lncRNA transcripts in three comparison groups during the implantation window Differential expression analysis was performed using the Cuffdiff program basing on FPKM value derived from Cufflinks, and *p*-values less than 0.05 were considered as significantly differentially expressed between two groups.

Based on *p*-values of < 0.05, there were 58 differentially expressed lncRNAs (60 transcript isoforms) in D4U samples relative to those in D5IU samples, of which 44 were significantly up-regulated and 14 (16 transcript isoforms) were down-regulated (Table 2 and Supplementary Table 1). Furthermore, a total of 35 lncRNAs (39 transcript isoforms) were differentially expressed based on pairwise comparisons between the D4U and D5NU samples with 29 (31 transcript isoforms) up-regulated and 6 (8 transcript isoforms) down-regulated. Overall, compared to implantation and inter-implantation sites of the day 5 pregnant mice, there were 69 differentially expressed lncRNA transcripts on day 4 of pregnancy with 50 significantly up-regulated and 19 significantly down-regulated (Figure 2 and Supplementary Table 1). A total of 6 (7 transcript isoforms) significantly up-regulated and 3 down-regulated differentially expressed lncRNAs were obtained in D5IU and D5NU comparison samples, respectively. As shown in Figure 2, just one of the differentially expressed lncRNA transcript (*Ensmust00000181242.1*) was common among the three groups. In addition, among the differentially expressed lncRNA transcripts examined, *Ensmust00000155046.1*, *Tcons_04171410*, *Tcons_01542665* and *Tcons_03121533* were only identified at day 4 and *Tcons_02454847* was only identified at the implantation site (Supplementary Table 1).

**Table 2:** Number of differentially expressed lncRNAs in three comparison groups during the implantation window

	D4U vs D5IU	D4U vs D5NU	D5IU vs D5NU
Up regulated	44 (44 transcripts)	29 (31 transcripts)	6 (7 transcripts)
Down regulated	14 (16 transcripts)	6 (8 transcripts)	3 (3 transcripts)
Total	58 (60 transcripts)	35 (39 transcripts)	9 (10 transcripts)

### The cis and trans target genes of lncRNAs

To investigate the function of lncRNAs, we first predicted the putative lncRNA *cis-* and *trans-*regulatory target genes. For the *cis* targets of lncRNAs, we considered the protein-coding genes located within 100 kb upstream and downstream, respectively, of the lncRNAs as *cis* target genes (Supplementary Table 2). The *trans* target genes located distant to lncRNAs are shown in Supplementary Table 3.

### Functional analysis of differentially expressed lncRNAs during the implantation window

**Figure 3:**
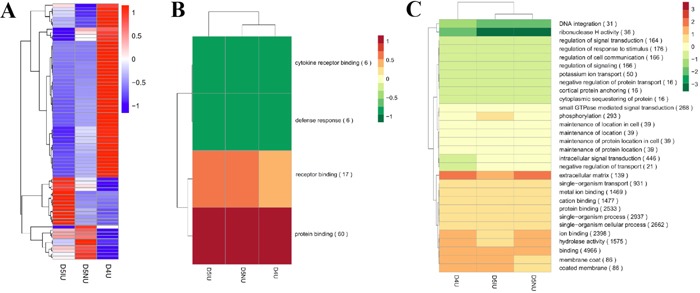
Hierarchical clustering analysis of differentially expressed lncRNAs in mouse uterus during the implantation window **(A)** The heat map of differentially expressed lncRNAs in the three comparison groups. Red indicates higher expression and blue indicates lower expression. Enrichment analysis of GO terms for differentially expressed lncRNAs in *cis* **(B)** and *trans* **(C)** from the three comparison groups. Red and green show higher and lower expression, respectively.

The heat map from all the differentially expressed lncRNAs in uterus tissue of D4-D5 pregnant mice clearly suggested that D5IU and D5NU were initially clustered together because their expression profiles were similar (Figure 3A). To further predict the function of lncRNAs during the implantation window, Gene Ontology (GO) and Kyoto Encyclopedia of Genes and Genomes (KEGG) analysis with the *cis* and *trans* lncRNA target genes in the three comparison groups were performed, respectively. Through GO analysis of *cis* lncRNA targets, we found that there was no significant GO terms enrichment in the D4U vs D5NU groups (corrected *p*-Value < 0.05, Figure 3B and Supplementary Table 4). The significantly enriched GO terms of *cis* lncRNA targets in the D5IU vs D5NU groups, which represent biological processes and molecular functions, were associated mainly with protein binding, receptor binding, defence response and cytokine receptor blinding. Cytokine receptor blinding was also significantly detected in the D4U vs D5IU groups. Furthermore, GO terms with the highest number of differentially expressed lncRNAs in the three comparison groups were involved mainly in tissue remodelling and metabolism and included cellular process, binding, catalytic activity, cellular metabolic process, organic substance metabolic process, metabolic process and single-organism process. In addition, KEGG analysis revealed that the significantly enriched *cis* pathways active during the implantation window were “Regulation of autophagy”, “RIG-I-like receptor signalling pathway”, “Cytosolic DNA-sensing pathway”, “Cytokine-cytokine receptor interaction”, “Natural killer cell mediated cytotoxicity”, “Jak-STAT pathway”, “PI3K-Akt pathway” and “Toll-like receptor pathway”, respectively (corrected *p*-Value < 0.05, Supplementary Table 5). Furthermore, the majority of lncRNA targets in *cis* are often implicated in “Ras pathway”, “Ubiquitin mediated proteolysis” and “Ribosome” in the three comparison groups (Supplementary Figure 3A).

Functional analysis illustrated that the *trans* lncRNAs targets were enriched in more than 3300 GO terms, encompassing a variety of biological processes (Supplementary Table 4). The GO terms with the highest number of *trans* targets were enriched mainly in the biological functions binding, protein binding, ion binding, single-organism process, single-organism cellular process and hydrolase activity (Figure 3C). Importantly, the significant enrichment GO terms of *trans* lncRNA targets were similar to the above top GO terms (Supplementary Table 4). The significant and most commonly enriched pathways of *trans* targets were related mainly to “Protein processing in endoplasmic reticulum”, “Pathways in cancer”, “DNA replication”, “RNA transport”, “Acute myeloid leukaemia”, “Proteoglycans in cancer”, “Cell cycle”, “Lysine degradation”, “Spliceosome”, “Ribosome biogenesis in eukaryotes”, “Metabolic pathways”, “Ras signalling pathway” and “Hippo signalling pathway” (Supplementary Figure 3B and Supplementary Table 5).

### Functional analysis of lncRNAs significantly up-regulated and down-regulated during the implantation window

To further reveal the specific function of significantly up-regulated and down-regulated lncRNAs in *cis* and *trans*, GO and KEGG analysis, respectively, were also conducted. There were no significantly enriched GO terms of the *cis* up-regulated lncRNAs in the D4U vs D5NU and D5IU vs D5NU comparison groups (corrected *p*-Value < 0.05, Supplementary Table 6). The GO terms significantly enriched in the D4U vs D5IU group were cytokine receptor binding and sequence-specific DNA binding. In addition, the most frequent GO terms of the up-regulated lncRNAs targets in *cis* from the three comparison groups were focused on immune function, cellular component biogenesis and tissue remodelling. KEGG analysis of *cis* up-regulated lncRNAs targets in the D4U vs D5IU group demonstrated that 18 significant pathways were involved mainly in the regulation of gene expression, but there was no significant pathway enrichment in *cis* for other two comparison groups (Supplementary Table 7). Interestingly, there were no significantly enriched pathways of up-regulated lncRNAs in *trans* for the D4U vs D5NU and D4U vs D5IU comparisons groups, but the most common in *trans* pathway enrichment groups was the same, including “Metabolic pathways”, “Pathways in cancer”, “PI3K-Akt signalling pathway”, “MAPK signalling pathway”, “Cytokine-cytokine receptor interaction” and “HTLV-I infection”. Furthermore, the significantly enriched pathway of D5IU vs D5NU groups in *trans* were related mainly to “RNA transport”, “DNA replication”, “Ribosome biogenesis in eukaryotes” and “Cell cycle.”

Because of the small number of significantly down-regulated lncRNAs in the three comparison groups, no significantly enriched GO terms in *cis* were observed, and the three most enriched GO terms were cellular process, binding and metabolic process (Supplementary Table 8). Through KEGG analysis, we found that the *cis* targets of significantly down-regulated lncRNAs were associated with metabolism, biosynthesis, and immune response (Supplementary Table 9). The maximum significantly enriched in *trans* GO terms of significantly down-regulated lncRNAs were involved mainly in the regulation of molecular function and biological process (Supplementary Table 8). There was no significant pathway enrichment in *trans* for the D4U vs D5NU and D5IU vs D5NU comparison groups (Supplementary Table 9). The significantly enriched pathway of D4U vs D5IU groups in *trans* were “RNA transport”, “DNA replication”, “Cell cycle”, “Ribosome biogenesis in eukaryotes” and “Protein processing in endoplasmic reticulum”. In addition, the maximum enriched pathway in *trans* for the three comparison groups included “Metabolic pathways”, “Pathways in cancer”, “PI3K-Akt signalling pathway”, “Ras signalling pathway”, and “MAPK signalling pathway”.

### qRT-PCR confirmation

**Figure 4:**
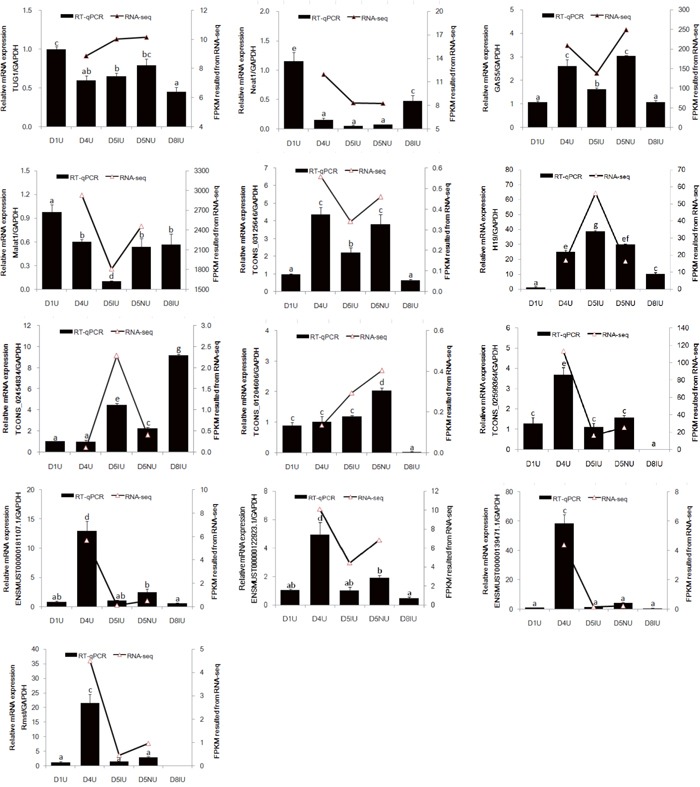
qRT-PCR validation of 13 differentially expressed lncRNAs in RNA-seq data The mouse uterine sample on pregnant day 1 (D1U), day 4 (D4U), day 5 at implantation sites (D5IU) and inter-implantation sites (D5NU) and day 8 at implantation sites (D8IU) were collected, respectively. LncRNAs expression was normalized with GAPDH using the 2^−ΔΔCt^ analysis method. The data are expressed as the mean ± S.E.M. from three replicates, and the bar bearing different superscript indicates a significant difference between the mean values (*P* < 0.05).

To validate the RNA-seq results, 13 selected lncRNAs, 9 known lncRNAs and 4 novel lncRNAs, were measured using qRT-PCR (Figure 4 and Supplementary Table 10). Regarding FPKM values, there was no significant difference in Taurine upregulated gene 1 (*Tug1*) and Nuclear Enriched Abundant Transcript 1 (*Neat1*) expression levels on days 4 and 5 of pregnancy. Interestingly, *Neat1* mRNA expression in day 1 and day 8 pregnant mouse was significantly higher than those from implantation window stage mice (*P* < 0.05). Growth arrest-specific 5 (*Gas5*), metastasis-associated lung adenocarcinoma transcription 1 (*Malat1*) and *Tcons_03125646* levels at the implantation site were significantly down-regulated during the implantation window (*P* < 0.05), although higher levels of *Gas5* and *Tcons_03125646* were observed at the implantation site compared to day 1 of pregnancy (*P* < 0.05). The expression levels of *H19* and *Tcons_02454834* were significantly up-regulated on day 5 at the implantation site during the implantation window (*P* < 0.05). Furthermore, the highest expression level of *Tcons_02454834* mRNA was observed in day 8 pregnant mouse uteri (*P* < 0.05). Surprisingly, the *Tcons_01204606* mRNA expression level increased dramatically on day 5 at the inter-implantation site and then was significantly down-regulated on day 8 of pregnancy (*P* < 0.05). Compared to days 1 and 5 of pregnancy, the expression levels of *Tcons_02599364*, *Ensmust00000181107.1*, *Ensmust*00000122923.1, *Ensmust00000139471.1* and rhabdomyosarcoma 2 associated transcript (*Rmst*) in day 4 pregnant mouse uteri were significantly up-regulated (*P* < 0.05). Furthermore, *Rmst* was not detectable in day 8 uteri. Taken together, the qRT-PCR validation results showed that the expression patterns of these lncRNAs were in excellent agreement with the RNA-seq data.

## DISCUSSION

Embryo implantation is a highly sophisticated and multifactorial process. A better understanding of the synchronized and successful molecular dialogue between the activated blastocyst and receptive endometrium may improve the ability to understand causes of pregnancy failures [25]. To date, several studies using genome-wide analysis have revealed many up- and down-regulated protein-coding genes at the implantation and inter-implantation sites in whole mouse uterine tissue or luminal epithelium during embryo implantation [26, 27]. As recent studies have revealed, a substantial number of lncRNAs exist in the mouse reproductive tract and play important functional roles in spermatogenesis, testis development, oocyte and embryonic development, trophoblast cell migration and invasion, sex determination and other reproductive processes [9, 15, 18, 28–31]. In the current study, 7,764 putative lncRNA transcripts were obtained from pregnant mouse uteri during the implantation window according to the RNA-seq data, and 1,589 of these were identified as novel lncRNAs. The biggest proportion (42.76%) of the lncRNAs was identified as lincRNAs, which do not overlap with other genes, suggesting that active transcription takes place mainly within intergenic regions in the mouse uterus during embryo implantation. Similar to previous studies, the putative lncRNAs identified in the present study have lower expression levels, lower conservation, shorter transcript length, smaller exon number and shorter ORF length compared to those of protein-coding genes.

The microarray analysis of luminal epithelium suggested that the majority of protein-coding genes expressed at the implantation sites were mainly related to extracellular matrix and tissue remodelling and others such as cell cycling, gene/protein expression, immune responses, invasion, metabolism, oxidative stress, or signal transduction [32]. In the present study, we found that the significant enrichment and higher number of GO terms in the *cis* and *trans* lncRNA targets from the three comparison groups were involved mainly in tissue remodelling and metabolism. It is known that the morphological changes of the pregnant uterus have important functional significance in the successful establishment of endometrial receptivity, which is tightly associated with the ultrastructural transformations of luminal epithelial cells, change in endometrial glands morphology and stromal cell differentiation [33]. This indicates that these lncRNAs may play an important role in endometrial remodelling during the implantation window. Furthermore, functional analysis of the *cis* lncRNAs targets at the implantation sites were also significantly enriched in the defence response, indicating that lncRNAs were involved in the immune response by regulating the neighbouring protein-coding genes. We further found that all of the GO terms of up-regulated lncRNAs targets in *cis* were also related to immune function, cellular component biogenesis and tissue remodelling. Furthermore, KEGG analysis showed that these *cis* differentially expressed lncRNAs were significantly enriched in several immune system-related pathways, such as RIG-I-like receptor signalling pathway, cytosolic DNA-sensing pathway, toll-like receptor signalling pathway, regulation of autophagy and natural killer cell mediated cytotoxicity. The first three of the above pathways play an important role in activating the innate immune response, which is necessary for successful embryo implantation and maintenance of pregnancy [34, 35]. In addition, the cytokine-cytokine receptor interaction, Jak-STAT signalling pathway and PI3K-Akt signalling pathway of lncRNA *cis* targets were also clearly identified and are known to be involved in the endometrial receptivity and embryo-endometrium interaction network in the implantation process [36, 37]. The results of the KEGG search suggested that the significantly and maximally enriched pathways of *trans* targets were involved in cell proliferation, differentiation, apoptosis, invasion and angiogenesis, including protein processing in endoplasmic reticulum, pathways in cancer, DNA replication, acute myeloid leukaemia, cell cycle, ribosome biogenesis in eukaryotes, Ras signalling pathway and so on. Similar results were also observed in the enriched pathway of up and down-regulated *trans* lncRNA targets. These findings suggested that lncRNAs act protein-coding genes in *trans* to regulate tissue structure and remodelling during the implantation window.

Compared with day 5 pregnant mouse uteri, there were 50 lncRNA transcript isoforms significantly up-regulated and 19 significantly down-regulated in day 4 pregnant mouse uteri. *Tcons_02599364*, *Rmst*, *Ensmust00000181107.1*, *Ensmust00000139471.1* and *Ensmust00000122923.1* were shown to be significantly up-regulated in day 4 pregnant mouse uteri by both RNA-seq and qRT-PCR results. Previous studies showed that *Rmst* is involved in the breast cancer and is functional in the embryonic dorsal forebrain and neurogenesis [38–40]. The Wnt/ß-catenin signalling is critical for *Rmst* expression, and the *Rmst*/*Micro-135a* expression pattern might play an important role in the Wnt/ß-catenin pathway and, possibly, the TGFβ/BMP pathway [41]. Both of Wnt/ß-catenin signalling and TGFβ/BMP pathway are best known for their critical roles in the changing of the endometrium before successful implantation. Interestingly, *Rmst* expression was not detected in day 8 pregnant mouse uteri, suggesting that *Rmst* might not be involved in uterine decidualisation. Although the mRNA levels of *Tug1* and *Neat1* in the mouse uterus from day 4 to 5 of pregnancy were not significantly different, *Tug1* and *Neat1* mRNA levels were significantly decreased compared with those of day 1 of pregnancy. *Tug1* acts as an important regulator in the development and progression of a variety of cancers, and downregulation of *Tug1* has been shown to inhibit cell proliferation, migration, invasion and promote apoptosis [42]. The process of embryonic implantation is strikingly similar to that of tumour cell development [43]. Moreover, the expression levels of *Tug1* showed a significant down-regulation in decidual endometrial stromal cells *in vitro* (unpublished data). *Neat1* knockout (KO) mice fail to establish successful pregnancy because of corpus luteum dysfunction and low serum progesterone levels [44]. *Neat1* KO mice also show aberrant mammary gland morphogenesis and lactation defects. *Neat1* localises villous trophoblast nuclei, and *Neat1* mRNA expression was up-regulated in placentas of mice undergoing intrauterine growth restriction (IUGR), indicating *Neat1* might play a role in placental dysfunction in idiopathic IUGR foetuses [45]. In the present study, the well-known lncRNAs *Gas5* and *Malat1* were also detected in the pregnant mouse uteri. The *Gas5* and *Malat1* mRNA levels were significantly down-regulated on day 5 at the implantation site during the implantation window. Consistent with the role in multiple cancers, *Gas5* expression has also been observed in human endometrial cells, human ovarian epithelium and mouse testis; however, the physiological significance of this expression is unclear [46–48]. *Malat1* was detected mainly in the nuclei of human endothelial cells, and its expression was higher in proliferative tissues compared secretory and post-menopausal endometrial tissues during the menstrual cycle [49]. In human and mouse endometrium, *H19* was expressed in stromal and myometrial cells, but not in luminal and glandular epithelium [50, 51]. Furthermore, *H19* expression was declined in the infertile women [52]. A recent study reported that *H19* promoted the proliferation of endometrial stromal cells through the *H19*/*Let-7*/*IGF1R* pathway [53]. In our study, *H19* was highly expressed in the pregnant mouse uteri during the implantation window compared with that of day 1 of pregnancy, and the highest peak of *H19* mRNA was detected on day 5 at the implantation site.

In conclusion, we present the first comprehensive annotation of lncRNAs in pregnant mouse uteri during the implantation window using deep RNA-seq analysis. Functional prediction of *cis* and *trans* factors suggested that the lncRNAs might play an important role in the regulation of protein-coding genes related to establishment of uterine receptivity. Our findings provide a valuable resource for further genomics research and functional studies of lncRNAs underlying embryo implantation.

## MATERIALS AND METHODS

### Ethics statement

All experiments involving animal procedures and variety of methods were approved by the Committee for the Ethics on Animal Care and Experiments at Northwest A&F University. All methods were performed in strict accordance with the Guidelines for the Committee for the Ethics on Animal Care and Experiments at Northwest A&F University. The mice were humanely sacrificed as necessary to ameliorate suffering.

### Animals and sample collection

Mature mice (Kunming White outbred strain) were obtained from the Laboratory Animal Center of Xi’An JiaoTong University. The mice were maintained in a temperature-(24 ± 2°C) and light-controlled room (12 h light:12 h darkness) with *ad libitum* access to food and water. Adult female mice were mated with fertile males of the same strain to induce natural pregnancy (day 1 is the day of vaginal plug). Pregnancy was ascertained on days 1 and 4 by recovering embryos from the oviduct and uterus, respectively. The implantation sites on day 5 of pregnancy were identified by intravenous injection of 0.1 ml of 1 % Chicago blue in saline (Sigma-Aldrich Co. LLC, Louis, MO, USA). On day 8, the whole uteri of pregnant mice were collected immediately after the mice were sacrificed by cervical dislocation. All samples were immediately snap frozen in liquid nitrogen and stored at -80°C until ready for RNA extraction.

### Total RNA isolation, library preparation and sequencing

Firstly, total RNA was isolated from the mouse uterus (three biological replicates per sample combined from five mice) using DeTRNa reagent (EarthOx, CA, USA) according to the manufacturer's protocol. Degradation and contamination of total RNA was monitored on 1% agarose gels. Purity and concentration of total RNA were measured using the NanoPhotometer® spectrophotometer (IMPLEN, CA, USA) and Qubit® RNA Assay Kit on the Qubit® 2.0 Fluorometer (Life Technologies, CA, USA), respectively. RNA integrity was assessed using the RNA Nano 6000 Assay Kit of the Bioanalyzer 2100 system (Agilent Technologies, CA, USA). Secondly, ribosomal RNA was removed from total RNA using Epicentre Ribo-zero™ rRNA Removal Kit (Epicentre, USA). Subsequently, sequencing libraries were generated using the rRNA-depleted RNA by NEBNext® Ultra™ Directional RNA Library Prep Kit for Illumina® (NEB, USA) according to the manufacturer's recommendations. To preferentially select cDNA fragments of 150~200 bp in length, the library fragments were purified with AMPure XP system (Beckman Coulter, Beverly, USA). Before PCR was performed, USER Enzyme (NEB, USA) was used with size-selected and adaptor-ligated cDNA. Lastly, PCR products were purified by AMPure XP system and library quality was assessed on the Agilent Bioanalyzer 2100 system. After clusters were generated using the TruSeq PE Cluster Kit v3-cBot-HS (Illumia) according to the manufacturer's instructions, the libraries were submitted to the Novogene Bioinformatics Technology Co., Ltd. (Beijing, China) and sequenced for 100 bp paired-end reads were sequenced on an Illumina Hiseq 2500 platform.

### Quality control and assembly of transcriptome data

Raw data in FASTQ format were first processed through in-house perl scripts. In this step, clean reads were obtained by removing reads containing adapters, reads containing poly-N and low quality raw reads. Furthermore, Q20, Q30 and GC content of the clean data were calculated. All the down-stream sequencing analyses were based on the high quality clean reads. For the RNA-seq data, all clean reads from each sample were aligned to the mouse reference genome ( ftp://ftp.ncbi.nlm.nih.gov/genomes/Mus_musculus/Assembled_chromosomes/seq/) using TopHat v2.0.9^49^. The distribution of known gene types was analysed by HTSeq software. The mapped reads of each sample were then assembled by both Scripture (beta2) [19] and Cufflinks (v2.1.1) [20] using a reference-based approach.

### Identification of candidate lncRNAs

The above assembled transcripts of all samples were combined with Cuffcompare software, and then the transcripts that were spliced by both Scripture and Cufflinks or appeared in at least two of the samples at the same time were selected; the transcripts ≥ 200bp and ≥ 2 exons were selected; the transcripts with a read coverage of ≥ 3 as calculated by cufflinks (v2.1.1) were further selected; the above transcripts were blasted with known mouse lncRNAs in GENCODE vM4 using Cuffcompare to filter the known lncRNAs; the lincRNA, intronic lncRNA and anti-sense lncRNA were identified from the remaining transcripts according to the results of class_code(http://cole-trapnell-lab.github.io/cufflinks/cuffcompare/index.html#transfrag-class-codes). Furthermore, to effectively filter the potential protein-coding transcripts, the basic filtering transcripts above were further analysed using four coding potential analysis software tools: CNCI (Coding-NonCoding-Index) (v2) [21], CPC (Coding Potential Calculator) (0.9-r2) [22], Pfam Scan (v1.3) [23] and PhyloCSF (phylogenetic codon substitution frequency) (v20121028) [24]. Transcripts predicted to have coding potential by any or all of the four above software tools were filtered out. Finally, the non-coding potential results from each software package that overlapped were identified as the candidate set of lncRNAs in our study.

### Classification of lncRNAs

According to their locations relative to the nearest protein-coding genes, the annotated lncRNAs were subdivided into four categories: (i) lncRNAs that do not overlap protein-coding genes, classified as lincRNAs; (ii) lncRNAs located entirely within a protein-coding locus, classified as intragenic lncRNAs; (iii) lncRNAs partially overlapping a protein coding gene, classified as overlapping-lncRNAs; and (iv) antisense lncRNAs overlapping exons of a protein-coding transcript on the opposite strand. Perl scripts were developed to classify these four categories.

### Quantification and differential expression analysis

The relative abundance of both candidate lncRNAs and coding genes in each sample was computed by calculating the FPKM using Cufflinks (v2.1.1). Differentially expressed lncRNAs in comparison groups were identified using the Cuffdiff program [20]. For biological replicates, transcripts with a *P*-adjust of < 0.05 were deemed differentially expressed between two groups.

### Predictions of cis and trans target genes

To explore the function of lncRNAs, we first predicted the *cis* and *trans* target genes of lncRNAs. We searched coding genes 10k/100k upstream and downstream, respectively, of candidate lncRNA as the *cis* target genes and then analysed their function. We calculated the expressed correlation between lncRNAs and coding genes with custom scripts and then analysed their function through functional enrichment analysis. The lncRNAs function in *trans* to identify each other by expression level.

### Conservative analysis

The PhyloFit programme from the Phast (v1.3) package was used to compute phylogenetic models for conserved and non-conserved regions among species, and then the model and HMM transition parameters were given to phyloP to compute a set of conservation scores of lncRNA and coding genes.

### GO and KEGG enrichment analysis

GO enrichment analysis of differentially expressed lncRNA target genes was implemented by the GOseq R package, in which gene length bias was corrected. In addition, KOBAS software and KEGG database (http://www.genome.jp/kegg/) were used to analyse the statistical enrichment of target genes of differential expression lncRNA in KEGG pathways. The lower the *P* value, the more prominent the relevance, and the corrected *p*-values of < 0.05 were considered significantly enriched by differential expressed genes.

### Validation of putative lncRNAs by qRT-PCR

Total RNA was isolated from uterine samples using Trizol reagent (TaKaRa Bio, Inc., Dalian, China). The RNA concentration and purity were measured by using a spectrophotometer (Eppendorf, Inc., Hamburg, Germany). The RNA integrity was determined by 1% agarose gel electrophoresis. cDNA was synthesized using the 5X All-In-One RT MasterMix with the AccuRT Genomic DNA Remove Kit (Applied Biological Materials Inc. BC, Canada) according to the manufacturer's instructions. qRT-PCR was performed with three biological replicates and technical triplicates/duplicates of each cDNA sample using the EvaGreen qPCR Mastermix Kit (Applied Biological Materials Inc. BC, Canada) with the CFX96™ Real-Time PCR Detection System (Bio-Rad Laboratories, Inc., Hercules, USA), according to the manufacturer's protocol. All primer information can be found in Supplementary Table 10. The relative levels of lncRNAs in each sample were normalized using GAPDH, and the lncRNA quantifications were performed using the 2^−ΔΔCt^ analysis method. The final data were presented as the mean ± SEM and analysed using an ANOVA followed by Fisher's least significant different test (Fisher LSD) with SPSS software (Version 13.0; SPSS, Inc., Chicago, IL). Differences were considered significant when *P* was < 0.05.

## SUPPLEMENTARY MATERIALS FIGURES AND TABLES





















## Abbreviations

E_2_: oestrogen
Gas5: Growth arrest-specific 5
GO: Gene Ontology
KEGG: Kyoto Encyclopedia of Genes and Genomes
lincRNAs: large intergenic non-coding RNAs
lncRNAs: long non-coding RNAs
Malat1: metastasis-associated lung adenocarcinoma transcription 1
Neat1: Nuclear Enriched Abundant Transcript 1
P_4_: progesterone
Rmst: rhabdomyosarcoma 2 associated transcript
Tug1: Taurine upregulated gene 1
