# Supplementary material for: Genome-wide analysis and functional prediction of long non-coding RNAs in mouse uterus during the implantation window
Source: Oncotarget. 2017 Sep 16;8(48):84360–72. doi: 10.18632/oncotarget.21031 (PMC5663602; doi:10.18632/oncotarget.21031)
Supplement: Supplementary file 1 [file oncotarget-08-84360-s001.pdf]

Genome-wide analysis and functional prediction of long non-coding RNAs in mouse uterus during the implantation window

SUPPLEMENTARY MATERIALS

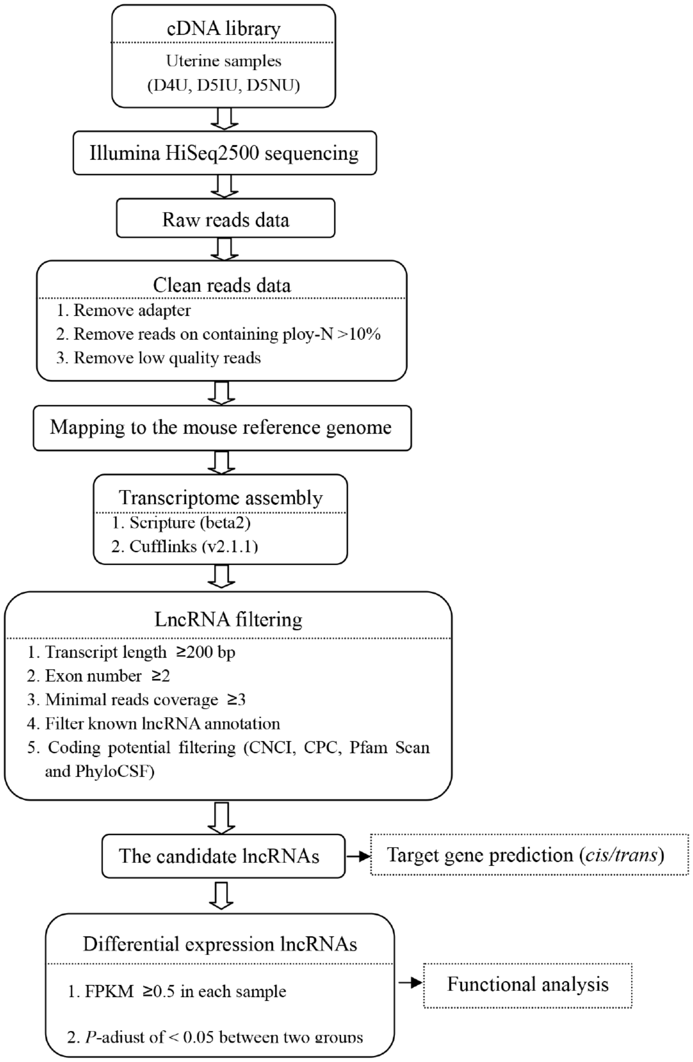

Supplementary Figure 1: Pipeline for identification of lncRNA by RNA-sequencing. Total RNA from the mouse pregnant uterus on day 4 (D4U), day 5 at implantation sites (D5IU) and day 5 at inter-implantation sites (D5NU) was isolated, respectively. After the quality of total RNA was assessed, ribosomal RNA was removed from total RNA and cDNA libraries were generated. Then, the libraries were sequenced on an Illumina Hiseq 2500 platform and raw reads data in FASTQ format were obtained. Clean reads data from each sample were generated by removing reads containing ploy-N >10% and low quality raw reads. All of above clean reads were aligned to the mouse reference genome using TopHat v2.0.949. The mapped reads were then assembled by both Scripture (beta2) and Cufflinks (v2.1.1). For identifying of candidate lncRNAs, the above assembled transcripts were combined with Cuffcompare software, and then the transcripts that were spliced by both Scripture and Cufflinks or appeared in at least two of the samples at the same time were selected. Furthermore, the above transcripts were further filtered according to the basic characteristics of lncRNA including transcript length ≥ 200bp, exon number ≥ 2 and non-coding protein. Finally, the non-coding potential transcripts predicted by all of the four software tools were identified as the candidate set of lncRNAs in our study. The FPKM ≥0.5 of above candidate lncRNAs in each sample were selected to obtain the differential expression profiles and the lncRNA transcripts with a *P*-adjust of < 0.05 between any two groups were identified as the differentially expressed lncRNAs.

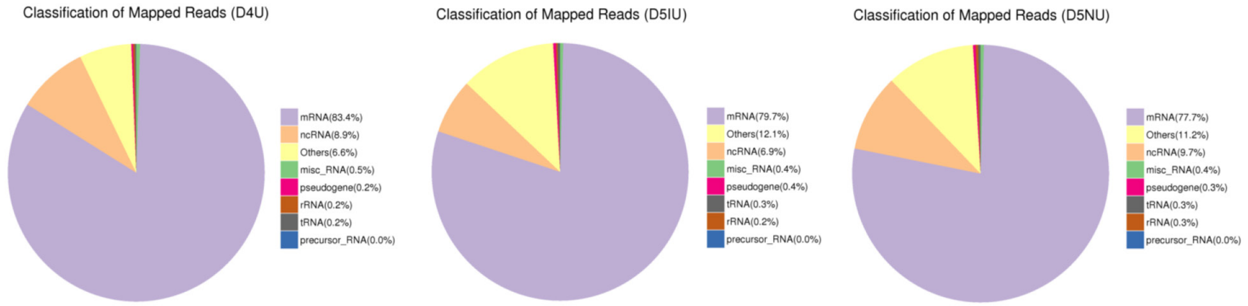

Supplementary Figure 2: Subtype distribution of the mapped reads according to genomic overlap with existing annotations using the HTseq software.

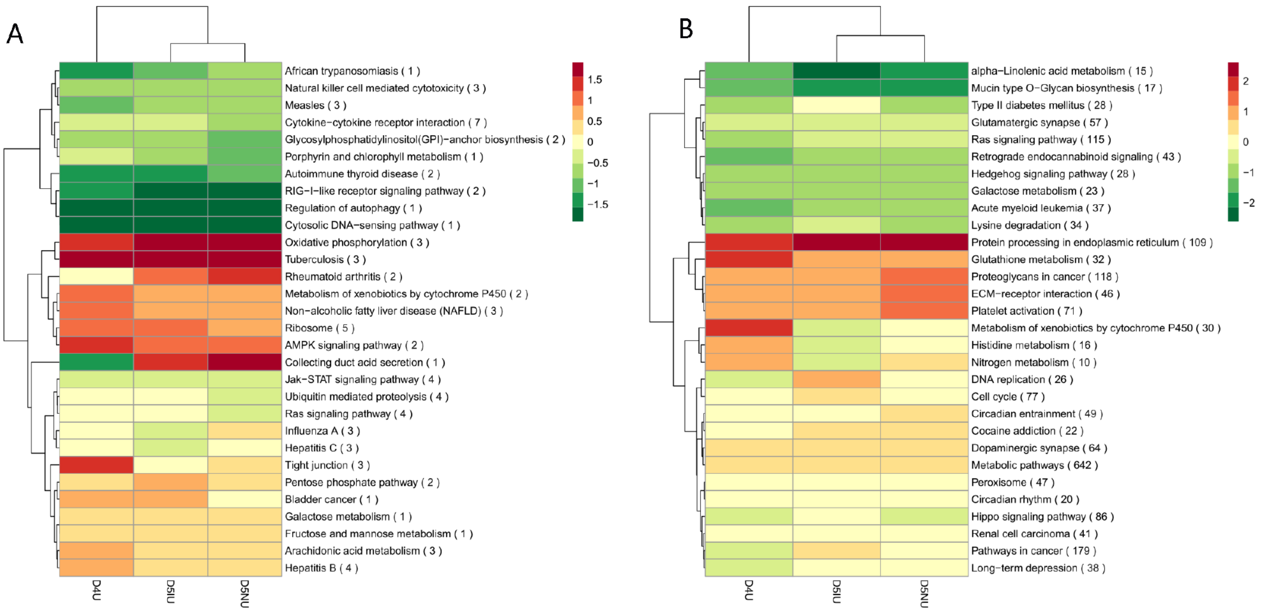

Supplementary Figure 3: Cluster analysis of KEGG pathways for differentially expressed lncRNAs in the three comparison groups.

Supplementary Table 1: The identified known and novel lncRNAs by RNA sequencing in mouse uterus during implantation window

See Supplementary File 1

Supplementary Table 2: The *cis* target genes of putative lncRNAs

See Supplementary File 2

Supplementary Table 3: The *trans* target genes of known lncRNAs and novel lncRNAs

See Supplementary File 3

Supplementary Table 4: Gene ontology analysis with the *cis* and *trans* lncRNA targets in three comparison groups

See Supplementary File 4

Supplementary Table 5: KEGG analysis with the *cis* and *trans* lncRNA targets in three comparison groups

See Supplementary File 5

Supplementary Table 6: Gene ontology analysis with the significantly up-regulated lncRNAs in three comparison groups

See Supplementary File 6

Supplementary Table 7: KEGG analysis with the significantly up-regulated lncRNAs in three comparison groups

See Supplementary File 7

Supplementary Table 8: Gene ontology analysis with the significantly down-regulated lncRNAs in three comparison groups

See Supplementary File 8

Supplementary Table 9: KEGG analysis with the significantly down-regulated lncRNAs in three comparison groups

See Supplementary File 9

Supplementary Table 10: Primer information of the selected lncRNAs used for validations by qRT-PCR

Gene name/transcript ID	Primer sequence	Product size (bp)	Tm (°C)
Tug1	F-CTCTGGAGGTGGACGTTTTGT<br>R-GTGAGTCGTGTCTCTCTTTTCTC	74	60
Neat1	F-GCTCTGGGACCTTCGTGACTCT<br>R-CTGCCTTGGCTTGGAAATGTAA	120	60
Gas5	F-GGAAGCTGGATAACAGAGCGA<br>R-GGTATTCCTTGTAATGGGACCAC	73	60
Rmst	F- AGCTGAATGTCTTTGTCCGC<br>R-GCGAGGACTGATGCCAATTT	204	60
Malat1	F-CATGGCGGAATTGCTGGTA<br>R-CGTGCCAACAGCATAGCAGTA	209	60
H19	F-GAACAGAAGCATTCTAGGCTGG<br>R-TTCTAAGTGAATTACGGTGGGTG	106	60
TCONS_03125646	F-GTGCTGTTCACCCTTTCCTC<br>R-GGCTGTGTGGTCTGAGTCTTC	131	60
TCONS_02454834	F-AGGTCTCTGGGCTTCAGGTC<br>R-CTGTGTGTGTCCTTCCACCA	115	60
TCONS_01204606	F-GAGGGATTGTTCGTTTCCAA<br>R-TTCCAGCAACTCTGTGGGTA	113	60
TCONS_02599364	F-ACGGTCTGTCCCATGATCTC<br>R-GGGACGAGAGACAGAGACAC	156	60
ENSMUST00000181107.1	F-CCCATGACAGACCAGAGTGT<br>R-TCTTTTGCCTTCCACAGCAC	216	60
ENSMUST00000139471.1	F-AGCTGCTCTCGTCTCTGAAG<br>R-GTTGTGGTGGTGGTGGTTTT	182	60
ENSMUST00000122923.1	F-CCGGCAGTCTTTGATACACG<br>R-GGGGAGGTTTCAGGTCTCAA	209	60
GAPDH	F-AGGTCGGTGTGAACGGATTTG<br>R-TGTAGACCATGTAGTTGAGGTCA	123	60
